# Extraction and Analysis of Six Effective Components in *Glycyrrhiza uralensis* Fisch by Deep Eutectic Solvents (DES) Combined with Quantitative Analysis of Multi-Components by Single Marker (QAMS) Method

**DOI:** 10.3390/molecules26051310

**Published:** 2021-03-01

**Authors:** Ping Yu, Qian Li, Yanmei Feng, Sinan Ma, Yuying Chen, Guichen Li

**Affiliations:** 1 College of Agronomy, Gansu Agricultural University, Lanzhou 730070, China; yp18325316756@163.com (P.Y.); fym15693446892@163.com (Y.F.); msn1805428551@163.com (S.M.); chenyuying0125@163.com (Y.C.); 2 Gansu Provincial Key Laboratory of Aridland Crop Science, Lanzhou 730070, China; lguchen@163.com

**Keywords:** flavonoids, saponins, DES, QAMS, ultrasonic-assisted extraction

## Abstract

Deep eutectic solvents (DESs) are green organic solvents that have broad prospects in the extraction of effective components of traditional Chinese medicine. This work employed the quantitative analysis of multi-components by a single marker (QAMS) method to quantitatively determine the six effective components of glycyrrhizic acid, liquiritin, isoliquiritin apioside, liquiritigenin, isoliquiritin, and glycyrrhetinic acid in *Glycyrrhiza uralensis*, which was used for comprehensive evaluation of the optimal extraction process by DESs. First, Choline Chloride: Lactic Acid (ChCl-LA, molar ratio 1:1) was selected as the most suitable DES by comparing the extraction yields of different DESs. Second, the extraction protocol was investigated by extraction time, extraction temperature, liquid-to-material ratio, molar ratio, and ultrasonic power. The Box–Behnken design (BBD) combined with response surface methodology (RSM) was used to investigate the optimal DES conditions. The result showed that the best DES system was 1.3-butanediol/choline chloride (ChCl) with the molar ratio of 4:1. The optimal extraction process of licorice was 20 mL/g, the water content was 30%, and the extraction time was 41 min. The comprehensive impact factor (z) was 0.92. At the same time, it was found that the microstructure of the residue extracted by the eutectic solvent was more severely damaged than the residue after the traditional solvent extraction through observation under an electron microscope. The DES has the characteristics of high efficiency and rapidity as an extraction solution.

## 1. Introduction

*Glycyrrhiza uralensis* Fisch is the dry root of the licorice, which belongs to the family Leguminosae. This plant is recognized as an important drug in the world and has been widely cultivated since ancient times [1]. *Glycyrrhiza uralensis* Fisch has Anti-inflammatory and antiviral effects in traditional and folk medicine [2]. Furthermore, *Glycyrrhiza uralensis* Fisch extract has anti-inflammatory, anti-viral, liver protection, detoxification and anti-aging effects in modern studies [3,4,5,6,7], so it is widely used in the treatment and prevention of diseases [8,9,10]. In the fight against the COVID-19, *Glycyrrhiza uralensis* Fisch was used well in the early stage of the COVID-19 [11,12]. The main components of *Glycyrrhiza uralensis* Fisch are flavonoids and saponins, among which flavonoids mainly have effects related to immune regulation [13], anti-cancer [14], anti-arrhythmia [15], anti-hepatic virus [16], and so on. In addition to medicinal usage, glycyrrhetinic acid and liquiritigenin in *Glycyrrhiza uralensis* Fisch are also used as a cake additive in food; its sweetness is one hundred times that of sucrose. It also has applications in cosmetics, tobacco, animal husbandry, etc. On the other hand, saponins have a variety of biological activities, such as anti-tumor, anti-inflammatory, immunomodulatory, anti-viral, anti-fungal, and hepatoprotective activities [17]. Therefore, the requirements for quality control and extraction rate of licorice are higher. In this article, a method of combining deep eutectic solvents (DESs) extraction and the quantitative analysis of multi-components by single marker (QAMS) method was established to realize the efficient evaluation of *Glycyrrhiza uralensis* Fisch.

QAMS can realize the simultaneous quantification of multiple components through the determination of one component. The multi-index quality evaluation model is better than a single index, which meets the characteristics of multi-components and multi-effect of traditional Chinese medicine (TCM) [18]. It has gradually been recognized by TCM industry, and has been applied to a certain range in the quality evaluation research and daily quality supervision of TCM [19,20,21,22]. In this study, glycyrrhizic acid was used as an internal standard to simultaneously quantify liquiritin, isoliquiritin apioside, liquiritigenin, isoliquiritin, and glycyrrhetinic acid to achieve the purpose of quality control. After research, it was found that the six components are high in content, easy to separate, and play an important role in the clinical application of *Glycyrrhiza uralensis* Fisch.

As known to us, it was found that the main extraction methods of *Glycyrrhiza uralensis* Fisch active ingredients were solvent extraction and steam distillation. Both of these methods have the disadvantages of low extraction efficiency and are likely to cause a waste of *Glycyrrhiza uralensis* Fisch resources [23]. DES is a new type of solvent used in recent years. It is environmentally friendly, easily degraded, and has high bioavailability [24]. It has a wide range of applications in the extraction of effective ingredients from plant materials [25,26,27,28,29,30,31]. In this work, the QAMS method is used to study the influence of eutectic solvent types, material-to-liquid ratio, ultrasonic power, ultrasonic temperature, and ultrasonic time on the extraction of six effective ingredients in *Glycyrrhiza uralensis* Fisch. It can be used for the research and development of *Glycyrrhiza uralensis* Fisch extraction technology to provide a theoretical basis and technical reference.

## 2. Results and Discussion

### 2.1. The f_m/k_ of Five Components

The *f_m/k_* was calculated by different injection volume, and the relative standard deviation (RSD) values were calculated as shown in Table 1. The effect of three different chromatographic columns, different column temperatures, and different volumetric flows on *f_m/k_* are shown in the Supplementary Materials (Tables S1–S3).

The content of the six components in the samples determined by QAMS and external standard method (ESM) were compared, and the error was expressed by the relative error (RE). RE% = [(QAMS − ESM)/EMS] × 100%. The results are shown in Table S4. The relative average deviation of the content of each component measured by the two methods is less than 5%, which indicates that the method is reliable.

### 2.2. Screening of Deep Eutectic Solvents

**Figure 1:**
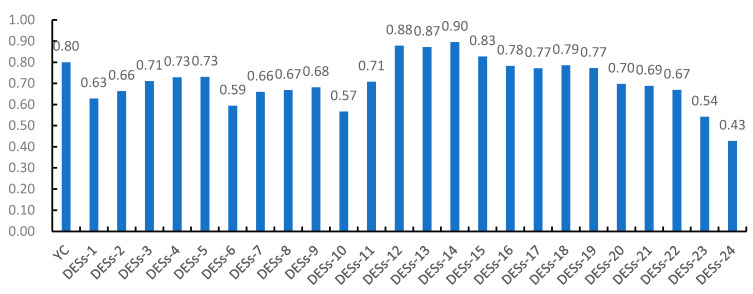
Comprehensive score using different types of deep eutectic solvents (DESs).

It was found that the number of hydrogen bonds, polarity, surface tension, and viscosity will have different effects on the components. The results are shown in Figure 1, after investigating 24 groups of different eutectic systems. As shown in Figure 1, it could be found that the differences were not only in the extraction rate of the effective components of *Glycyrrhiza uralensis* Fisch by different DESs systems but also between different molar ratios of the same system. Therefore, 1,3-butanediol/ChCl (molar ratio 4:1) was selected as the extraction solvent for the extraction of isoliquiritin apioside, liquiritin, isoliquiritin, liquiritigenin, glycyrrhizic acid, glycyrrhetinic acid from the *Glycyrrhiza uralensis* Fisch.

As we all know, the extraction efficiency of compounds was affected by many factors, such as temperature, humidity, surface area, polarity, viscosity, diffusion, and so on. It was shown that DESs increase their solubility mainly through the formation of intermolecular hydrogen bonds and electrostatic interaction. This experiment discussed 24 combinations of DESs. DES1-15,24 contain a large number of hydroxyl groups, DES21-22 contain amino groups, and DES16-20,23 contain carboxy groups and could build hydrogen bonds with flavonoids and saponin compounds with the six target compounds. Flavonoids contain multiple cyclopentene structures to form a π–π large conjugated system, which has a high electron cloud density [32,33,34]. The carboxyl gene C=O bond of DES has strong electronegativity and a high electron cloud density. When the two are close, the electron cloud will affect the stability of the hydrogen bond due to mutual repulsion; however, saponins do not have a π–π conjugated system, and there is no problem of electron cloud repulsion, and a strong electronegativity C=O is conducive to the formation of hydrogen bonds. Therefore, the comprehensive score of the target produced was determined by the type of DES. The comprehensive score of DES-14 for the target compound is higher, which may be due to the increased formation of hydrogen bonds and higher polarity. During the experiment, the viscosity of DES-14 was small, and the smaller viscosity was beneficial to increase the diffusivity. In short, DES-14 as an extraction solvent has a higher comprehensive score for the six components of licorice. There might be more complicated reasons that need to be explored.

### 2.3. Effects of Single Factor of DES

The influence of the water content on *Glycyrrhiza uralensis* Fisch was examined by examining the water content as follows: 10%, 20%, 30%, 40%, and 50%. The results are shown in Table 2. When the moisture content is 30%, the comprehensive score reached the highest level, so 30% moisture content was chosen as the initial extraction condition.

The effects of different liquid–material ratios of 10, 20, 30, 40, and 50 mL/g on the extraction of active ingredients of *Glycyrrhiza uralensis* Fisch were investigated. As shown in Table 2, the extraction effect was the best when the liquid–solid ratio is 20 mL/g.

In this study, the extraction times of 20, 30, 40, 50, and 60 min on the extraction rate of active ingredients of *Glycyrrhiza uralensis* Fisch were investigated. As shown in Table 2, the comprehensive score reached the highest when the extraction time is 40 min, so 40 min was chosen as the initial extraction condition.

The extraction temperature was chosen as 30, 40, 50, 60, 70, and 80 °C, when we investigated the effect of temperature on the extraction rate of the effective components of *Glycyrrhiza uralensis* Fisch. It is found that the comprehensive score was the highest when the extraction temperature is 50 °C (Table 2).

The effect of ultrasonic power of 240, 300, 360, 420, and 480 W on the extraction rate of effective components of *Glycyrrhiza uralensis* Fisch was investigated, and the extraction temperature was selected as 40 °C. It was found that the ultrasonic power of 300 W had the highest comprehensive score, which is shown in Table 2.

### 2.4. Influence of Different Extraction Methods on Microstructure

In order to study the microscopic effects of different extraction methods on *Glycyrrhiza uralensis* Fisch powder, four extraction methods were used to treat *Glycyrrhiza uralensis* Fisch powder separately. In Method 1, 100 mL of DES was used to extract *Glycyrrhiza uralensis* Fisch with the aid of ultrasound. In Method 2, 100 mL of 70% ethanol was used to extract *Glycyrrhiza uralensis* Fisch by ultrasonic reflux. In Method 3, 100 mL of DES was used to heat and reflux *Glycyrrhiza uralensis*.

**Figure 2:**
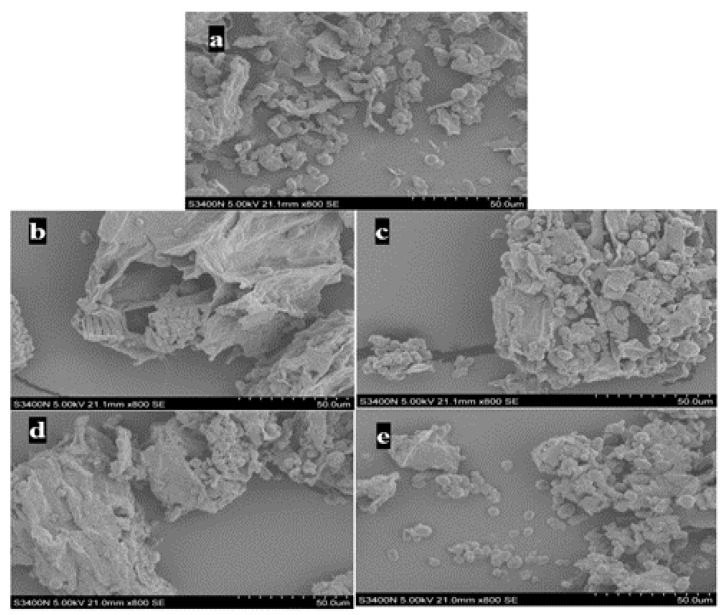
(**a**) *Glycyrrhiza uralensis* Fisch crude drug powder; (**b**) *Glycyrrhiza uralensis* Fisch powder obtained by ultrasonic-assisted extraction of DESs; (**c**) *Glycyrrhiza uralensis* Fisch powder obtained by ultrasonic-assisted extraction of ethanol; (**d**) *Glycyrrhiza uralensis* Fisch powder obtained by heating and refluxing extraction of DESs; (**e**) *Glycyrrhiza uralensis* Fisch powder obtained by heating and refluxing the ethanol solution.

Fisch powder for extraction. In Method 4, 100 mL of 70% ethanol was used to heat and reflux *Glycyrrhiza uralensis* Fisch powder for extraction. Scanning electron microscopy (SEM) was used to observe the residues of different extraction methods, which is shown in Figure 2.

As shown in Figure 2, it can be seen that the *Glycyrrhiza uralensis* Fisch residue cells obtained by DESs ultrasonic-assisted treatment of *Glycyrrhiza uralensis* Fisch powder were the most disrupted, so it can be seen that DESs has the highest treatment efficiency for *Glycyrrhiza uralensis* Fisch. This result was the same as the result shown in Section 2.2. The broken cells were proportional to the extraction rate. In this study, DESs ultrasonic-assisted treatments were selected as the best method of *Glycyrrhiza uralensis* Fisch.

### 2.5. Optimization of the Extraction Conditions

In this study, Box–Behnken design (BBD) combined with response surface methodology (RSM) was used to investigate the optimal DES conditions. On the basis of the single factor experiment results, three factors as independent variables, the liquid to material ratio (A), the water content (B), and extraction time (C) were investigated at three levels (Table 3). The comprehensive score value of liquiritin apioside, glycyrrhizin, isoliquiritin, glycyrrhizin, glycyrrhizin, and glycyrrhetinic acid (Z-score, weight coefficients are 1/n) were taken as the inspection index, and the eutectic solvent for *Glycyrrhiza uralensis* Fisch index into the best extraction process were optimized. The factor levels and experimental results are shown in Table 3.

**Figure 3:**
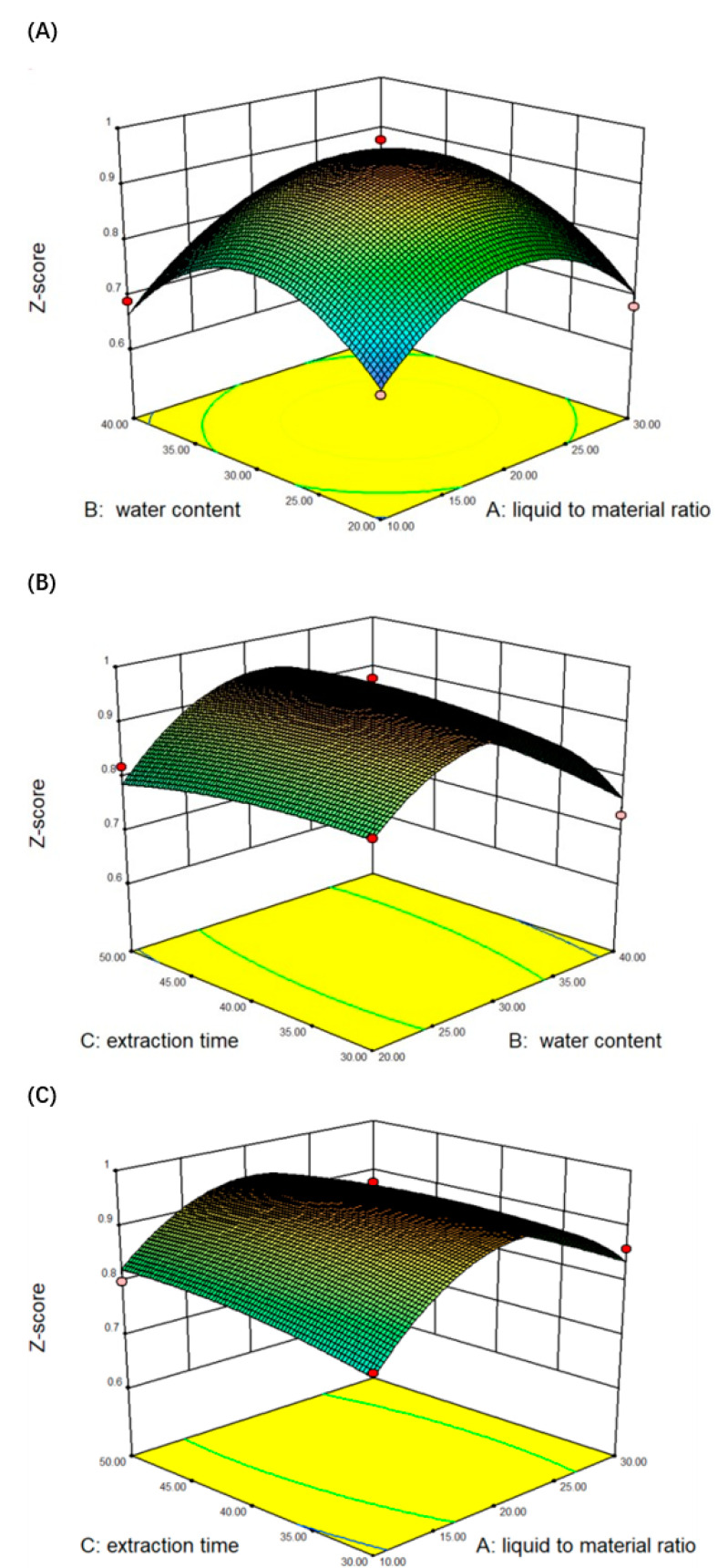
Comprehensive score response surface of active ingredients in *Glycyrrhiza uralensis.* Note: The interaction between liquid to material ratio and water content (**A**); The interaction between extraction time and liquid to material ratio (**B**); Interaction between extraction time and water content (**C**).

In this study, the extraction of six target compounds was optimized. The experimental design matrix and levels are shown in Table 4. Then, three factors and three levels were selected for BBD, and 17 experiments were performed in this study. The three-dimensional (3D) response surface analysis of multiple non-linear regressions used the comprehensive score of six target compounds as the response value. The 3D diagram of the interaction of the three factors is shown in Figure 3A–C. The predicted values of the responses were obtained from a quadratic model to the following equations:

Z-score = + 0.95 + 0.013A − 0.0075B + 0.005C + 0.005AB − 0.020AC + 0.025BC − 0.13A^2^ − 0.14B^2^ − 0.016 C^2^ (1)

where A is the ratio of liquid to material, B is the water content, and C is the extraction time. Z is the comprehensive score of the six active ingredients.

Table 5 shows the ANOVA for the quadratic models of the comprehensive score for the six effective ingredients of *Glycyrrhiza uralensis*. According to ANOVA analysis, A^2^ < 0.01 and B^2^ < 0.01 indicated that the impact was very significant.

It could be found that the model *p* = 0.0003 < 0.01 (Table 5), indicating that the model was extremely significant, the lack of fit term *p* = 0.0612 > 0.05 is not significant, the *R^2^* of regression equation was 0.9657, the corrected coefficient R^2^ was 0.9216, indicating that the linear relationship between the independent variable and the dependent variable was significant, which can be used to optimize the extraction process of the effective components of *Glycyrrhiza uralensis* Fisch.

As shown in Figure 3, the interaction of the liquid-to-material ratio (A), water content (B), and extraction time (C) has a significant impact on the comprehensive score of DESs extracted *Glycyrrhiza uralensis* active ingredients. Through the 3D graph, the slope determines the relationship between the image factors. The higher the curved surface, the steeper the slope. In addition, a preliminary judgment can be made from the color of the 3D image. As the change trend increased sharply, the color also showed a darkening trend, and the shape of the contour can also reflect the strength of the interaction effect. The shape of the contour line reflects the strength of the interaction effect. If the interaction between the two factors was significant, the contour line was elliptical.

The liquid/material was steeper than the three-dimensional response surface map of (a) and water content (b), indicating that its interaction has less influence on the liquid/material.

### 2.6. Method Validation

#### 2.6.1. Linearity, Limit of Detection (LOD), and Limit of Quantification (LOQ)

First, the appropriate amounts of reference substances apioside liquiritin, glycyrrhizin, isoliquiritin, glycyrrhizin, glycyrrhizin, and glycyrrhetinic acid were precisely weighed. Next, we placed them in 6 different 10 mL measuring flasks, added 70% ethanol to the mark, and obtained the reference solution (the mass concentration of each component is 0.43, 0.45, 0.26, 0.35, 0.56, and 0.22 mg/mL, respectively). Last, we accurately measured 1 mL of each standard solution, added 70% absolute ethanol to a 10 mL measuring flask, and diluted the volume to the mark to obtain a mixed reference solution (mass concentration of 0.043, 0.045, 0.026, 0.035, 0.056, and 0.022 mg/mL). Then, we divided the mixed solution into five injections (4, 8, 10, 16, 20 ul). According to the HPLC results of five different concentrations of reference substance, the function relationship between concentration (X) and peak area (Y) was obtained, as shown in Table 6. The R^2^ of liquiritin, glycyrrhizic acid, liquiritigenin, isoliquiritin, glycyrrhetinic acid, and isoliquiritin apioside were both greater than 0.999, indicating a good linear relationship. Table 6 shows the regression data for six bioactive compounds obtained by HPLC results.

#### 2.6.2. Precision, Repeatability, Stability, and Recovery

The standard mixed solution of *Glycyrrhiza uralensis* Fisch was taken and injected 6 times continuously according to the chromatographic conditions under “3.5.1”. The peak area values were recorded, and the RSD values were calculated. The RSD for isoliquiritin apioside, liquiritin, isoliquiritin, liquiritigenin, glycyrrhizic acid, and glycyrrhetinic acid results was 0.60%, 0.33%, 1.05%, 0.45%, 0.98%, and 1.02%, respectively, indicating that the instrument has good precision.

Six samples of the solution obtained under “3.1” were precisely injected to the HPLC system, the samples according to the chromatographic conditions under “3.5.1”. The RSD values of isoliquiritin apioside, liquiritin, isoliquiritin, liquiritigenin, glycyrrhizic acid, and glycyrrhetinic acid were 1.24%, 1.43%, 2.01%, 1.77%, 0.48% and 1.49%, respectively. Therefore, the method has good repeatability.

Then, 10 μL of the test solution obtained under “3.1” was analyzed under the chromatographic conditions under “3.5.1” at 1, 2, 8, 16, and 24 h. The results showed that the RSD values of the peak area of isoliquiritin apioside, liquiritin, isoliquiritin, liquiritigenin, glycyrrhizic acid, and glycyrrhetinic acid were 2.02%, 1.93%, 1.77%, 1.32%, 1.03%, and 2.39%, respectively. Therefore, the result indicated that the test solution has good stability within 24 h.

The recovery rate of standard addition = (measured value of spiked sample-measured value of sample) ÷ spiked amount × 100%. Thus, the sample solution with a known content was divided into three, and we doubled the amount for content determination. The sample recovery rates of isoliquiritin apioside, liquiritin, isoliquiritin, liquiritigenin, glycyrrhizic acid, and glycyrrhetinic acid were 102.12%, 104.22%, 101.38%, 103.41%, 103.36%, and 100.97%, respectively. The RSDs for isoliquiritin apioside, liquiritin, isoliquiritin, liquiritigenin, glycyrrhizic acid, and glycyrrhetinic acid were 1.43%, 0.03%, 0.58%, 0.57%, 0.41%, and 0.51% respectively.

### 2.7. Verification of Predictive Model

The optimal eutectic solvent extraction conditions for *Glycyrrhiza uralensis* Fisch were obtained by solved the extreme value of the model and analyzed the contours by the software. The liquid to material ratio was 20.40 mL/g, the water content was 29.84%, and the extraction time was 41.19 min. The comprehensive score under the best extraction process was 0.95. Combining single factor experiments and actual production considerations, the process conditions for the DES of *Glycyrrhiza uralensis* Fisch were 20 mL/g of liquid to material ratio, 30% water content, and 41 min extraction time, which can meet the extraction requirements. Therefore, three confirmatory experiments referring to the best solution obtained by response surface optimization were carried out, and the liquid-to-material ratio was 20, the water content was 30%, and the extraction time was 41 min. The overall score of the effective components of *Glycyrrhiza uralensis* Fisch was 0.92. The RSD was 0.50%. The specific extraction rate is shown in Table 7.

## 3. Materials and Methods

### 3.1. Plant Materials

*Glycyrrhiza uralensis* Fisch (20191010) was purchased from the Yellow River Medicinal Material Market Lanzhou (Lanzhou, China) and was identified as a piece of dried root and rhizome of the legume *Glycyrrhiza uralensis* Fisch by Professor Chen Yuan from the Department of Chinese Herbal Medicine of Gansu Agricultural University (Lanzhou, China). The remaining samples were stored in the Traditional Chinese Medicine Analysis Laboratory of Gansu Agricultural University (Lanzhou, China).

### 3.2. Chemicals and Reagents

Liquiritin (19082902, ≥98%), glycyrrhizic acid (19032704, ≥98%), liquiritigenin (17112302, ≥98%), isoliquiritin (20041301, ≥98%), and glycyrrhetinic acid (19051006, ≥98%) were purchased from Chengdu Pfeidian Biotechnology Co., Ltd. (Chengdu, China); isoliquiritin apioside (MUST-19123101, ≥98%) was purchased from Chengdu Mansite Biotechnology Co., Ltd. (Chengdu, China); urea, ethylene glycol, glycerol, and 1,3-butanediol are all Analytical Reagents; acetonitrile, phosphoric acid, and absolute ethanol are chromatographic pure; water is ultrapure water. The quality scores of each control were ≥98%.

### 3.3. Preparation of DESs

The synthetic methods used in this study are all according to the method reported by Abbota et al. [24,35,36]. Firstly, the appropriate molar ratio of hydrogen bond donors (HBDs) and hydrogen bond acceptors (HBAs) were put in a 250 mL conical flask. Secondly, the flask was heated and stirred at 80°C until a uniform and stable liquid was formed. Thirdly, the products were stored at room temperature. In this study, 24 different kinds of DESs solvents were investigated, as shown in Table 8.

### 3.4. Extraction Procedure

#### 3.4.1. Reference Extraction Methods

According to the Chinese Pharmacopoeia for extraction [37], 1.0 g (± 0.01 mg) *Glycyrrhiza uralensis* Fisch roots samples (passed through a 40-mesh sieve) were accurately weighed, and then a certain volume of DESs were put into a stoppered conical flask (250 mL) and ultrasonically treated 40 min (power 300 W, frequency 40 kHz). After the treatment, all extracts were filtered through 0.45 μm nylon membranes before HPLC analysis. All the experiments were performed in triplicate.

Then, 1.0 g (± 0.01 mg) of *Glycyrrhiza uralensis* Fisch roots samples were accurately weighed and different types of DESs with a water content of 30% were added, which were also compared with ethanol extraction.

#### 3.4.2. Preparation of Standard Solution

Firstly, the appropriate amounts of standard substances were accurately weighed and placed in the different 10 mL volumetric flasks with 70% ethanol to configure into solutions of different concentrations. The mass concentration of isoliquiritin apioside, liquiritin, isoliquiritin, liquiritigenin, glycyrrhizic acid, and glycyrrhetinic acid were 0.43, 0.45, 0.26, 0.35, 0.56, and 0.22 mg/mL.

### 3.5. Establishment of QAMS Method

#### 3.5.1. Quantitative Analysis of HPLC

**Figure 4:**
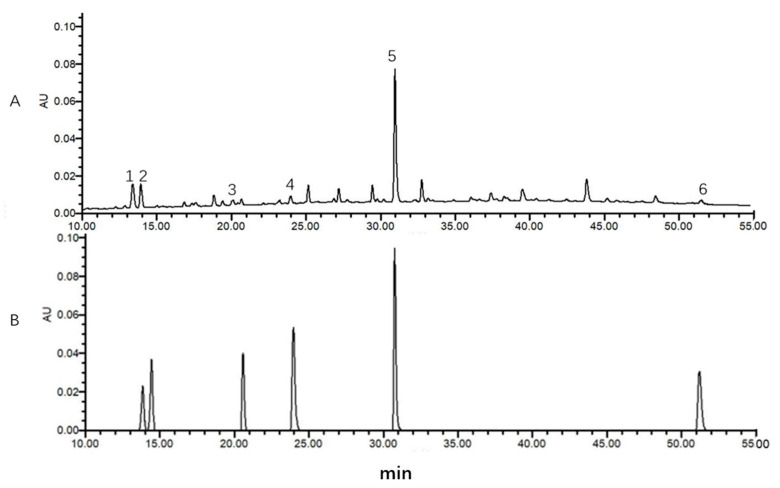
HPLC chromatograms of medicinal material extract (**A**) and mixed reference solution (**B**). Note: 1 is isoliquiritin apioside, 2 is liquiritin, 3 is isoliquiritin, 4 is liquiritigenin, 5 is glycyrrhizic acid, and 6 is glycyrrhetinic acid.

Quantitative HPLC analysis was performed on a Waters (ACQUITY ARC) chromatography system equipped with a Waters detector (2998PDA). The chromatographic column was a Waters Symmetry C1 8 (4.6 mm × 250 mm, 5 μm); the mobile phase was acetonitrile-0.005% phosphoric acid aqueous solution [38]. The gradient elution: 0–20 min, 0–12% acetonitrile, 20–45min, 12–32% acetonitrile; 45–75 min, 32–70% acetonitrile; 75–76 min, 70–98% acetonitrile; 76–80min, 97–12% acetonitrile; and the detection wavelength was set at 254 nm. The column was operated at 25 °C with the mobile phase at a constant flow rate of 1 mL/min. HPLC chromatograms of the standard compound and sample are presented in Figure 4.

#### 3.5.2. Relative Correction Factor (f_m/k_) Calculation

Mixed reference solution was drawn 4, 8, 12, 16, 20 μL and chromatographic analyzed according to the chromatographic conditions in Section 3.5.1. With glycyrrhizic acid as the internal standard substance, according to the formula *f_m/k_* = A_k_C_m_/A_m_C_k_ (A_k_ is the peak area of the internal standard substance, C_k_ is the mass concentration of the internal standard substance, A_m_ is the peak area of the other component “m”, and C_m_ is the mass concentration of other components “m”).

#### 3.5.3. Comprehensive Evaluation Index

We used QAMS and Box–Behnken design (BBD) for comprehensive evaluation of extraction efficiency. The weighted of each index were assigned as 1/n according to the pharmacological effects. Comprehensive score (Z) = (isoliquiritin apioside score + liquiritin score + liquiritin score + liquiritigenin score + glycyrrhizic acid score + glycyrrhetinic acid Score)/6. Index score = extraction rate of the index/highest extraction rate in the group of the index. The DES screening experiment, single factor experiment, and response surface experiment analysis of this experiment were all based on the results of comprehensive scoring. In the experiment, *f_m/k_* was used to calculate the content of isoliquiritin apioside, liquiritin, liquiritin, liquiritigenin, glycyrrhizic acid, and glycyrrhetinic acid with glycyrrhizic acid as the internal standard, and the calculation formula is as follows.


Amount in sample = *f_m/k_* × A_m_ × C_k_/A_k_ × 100/W (2)

*f_m/k_* is the relative correction factor of each component, A_m_ is the peak area of the other components “m”, A_k_ is the peak area of the internal standard substance, C_k_ is the concentration of the internal standard substance, and W is the sample weight.

### 3.6. Experimental Design

The single factor experimental conditions were optimized according to the ultrasound-assisted conditions in order to obtain appropriate extraction conditions, the liquid–material ratio of 1,3-butanediol to ChCl (10, 20, 30, 40, and 50 mL/g), the water content in 1,3-butanediol/ChCl (20, 30, 40, 50, and 60%), the extraction time (20, 30, 40, 50, and 60 min), the extraction temperature (30, 40, 50, 60, 70, and 80 °C), and the ultrasonic power (240, 300, 360, 420, and 480 W). The finally choices of liquid–material ratio, water content, and extraction time were the three factors of this experiment.

## 4. Conclusions

In this study, glycyrrhizic acid was used as the internal standard to construct a QAMS method and calculate the content of liquiritin, liquiritigenin, isoliquiritin, glycyrrhetinic acid, and isoliquiritin apioside, respectively. ChCl was used as the HBA and 1,3-butanediol was used as the HBD with a molar ratio of 1:4. The best liquid-to-material ratio optimized by the BBD-RSM experiment was 20 mL/g, the water content was 30%, the extraction time was 41 min, and the comprehensive score was 0.92. The combination of QAMS and the DESs extraction process for *Glycyrrhiza uralensis* Fisch showed that QAMS has good practical applications and could be used for the rapid and efficient evaluation of the quality of TCM. On the other hand, it showed that the natural green solvent DESs could be used as an efficient, feasible, stable, high-quality extraction solvent to extract the effective ingredients of *Glycyrrhiza uralensis* Fisch. Furthermore, this work provided a promising strategy to extract and analyze active compounds from Chinese herbal medicines.

## Figures and Tables

**Table 1:** The *f_m/k_* of five components with glycyrrhizic acid as internal standard.

Injection Volume/μL	*f_isoliquiritin apioside/glycyrrhizic acid_*	*f_liquiritin/glycyrrhizic acid_*	*f_isoliquiritin/glycyrrhizic acid_*	*f_liquiritigenin/glycyrrhizic acid_*	*f_glycyrrhetinic acid/glycyrrhizic acid_*
4	1.57	1.34	0.88	0.73	0.78
8	1.60	1.33	0.87	0.71	0.80
10	1.59	1.35	0.86	0.70	0.80
16	1.61	1.34	0.87	0.71	0.77
20	1.58	1.33	0.87	0.71	0.79
average	1.59	1.34	0.87	0.71	0.79
RSD	0.86%	0.51%	1.05%	1.66%	1.64%

**Table 2:** Extraction rate and comprehensive score of each index component under different single factors.

Single Factor	Factor Level	Extraction Rate/%					Z-Score	
		Isoliquiritin Apioside	Liquiritin	Isoliquiritin	Liquiritigenin	Glycyrrhizic Acid	Glycyrrhetinic Acid	
Water content/%	10	0.59 ± 0.0047	0.39 ± 0.0037	0.08 ± 0.0041	0.05 ± 0.0045	1.62 ± 0.0033	0.07 ± 0.0045	0.84
	20	0.59 ± 0.0065	0.41 ± 0.0042	0.08 ± 0.0044	0.04 ± 0.0044	1.7 ± 0.0040	0.07 ± 0.0042	0.83
	30	0.66 ± 0.0057	0.44 ± 0.0027	0.1 ± 0.0042	0.05 ± 0.0034	1.83 ± 0.0043	0.07 ± 0.0047	0.95
	40	0.61 ± 0.0036	0.42 ± 0.0045	0.12 ± 0.0045	0.03 ± 0.0027	1.72 ± 0.0034	0.05 ± 0.0040	0.83
	50	0.66 ± 0.0028	0.43 ± 0.0041	0.14 ± 0.0040	0.03 ± 0.0047	1.73 ± 0.0034	0.03 ± 0.0037	0.82
Liquid to Material ratio/mL·g^−1^	10	0.59 ± 0.0032	0.4 ± 0.0033	0.08 ± 0.0034	0.03 ± 0.0044	1.67 ± 0.0050	0.12 ± 0.0033	0.83
	20	0.64 ± 0.0041	0.44 ± 0.0047	0.1 ± 0.0045	0.05 ± 0.0047	1.83 ± 0.0034	0.07 ± 0.0043	0.91
	30	0.59 ± 0.0023	0.4 ± 0.0033	0.11 ± 0.0029	0.03 ± 0.0029	1.69 ± 0.0035	0.07 ± 0.0040	0.8
	40	0.62 ± 0.0034	0.42 ± 0.0025	0.09 ± 0.0050	0.05 ± 0.0047	1.71 ± 0.0029	0.06 ± 0.0035	0.83
	50	0.67 ± 0.0043	0.44 ± 0.0050	0.1 ± 0.0047	0.05 ± 0.0050	1.8 ± 0.0040	0.07 ± 0.0036	0.9
Extraction time/min	20	0.62 ± 0.0033	0.42 ± 0.0045	0.09 ± 0.0037	0.03 ± 0.0042	1.74 ± 0.0047	0.06 ± 0.0037	0.79
	30	0.62 ± 0.0042	0.44 ± 0.0034	0.1 ± 0.0050	0.05 ± 0.0043	1.87 ± 0.0045	0.07 ± 0.0043	0.9
	40	0.7 ± 0.0034	0.47 ± 0.0033	0.13 ± 0.0039	0.03 ± 0.0048	1.89 ± 0.0038	0.07 ± 0.0042	0.93
	50	0.6 ± 0.0035	0.42 ± 0.0044	0.11 ± 0.0038	0.03 ± 0.0036	1.74 ± 0.0039	0.06 ± 0.0043	0.82
	60	0.62 ± 0.0045	0.43 ± 0.0023	0.11 ± 0.0037	0.03 ± 0.0039	1.75 ± 0.0034	0.07 ± 0.0040	0.83
Extraction temperature/°C	30	0.63 ± 0.0044	0.42 ± 0.0045	0.11 ± 0.0045	0.03 ± 0.0036	1.78 ± 0.0039	0.04 ± 0.0045	0.77
	40	0.6 ± 0.0035	0.41 ± 0.0047	0.06 ± 0.0047	0.02 ± 0.0042	1.72 ± 0.0043	0.04 ± 0.0029	0.64
	50	0.67 ± 0.0043	0.43 ± 0.0042	0.13 ± 0.0049	0.03 ± 0.0037	1.92 ± 0.0032	0.12 ± 0.0034	0.93
	60	0.67 ± 0.0032	0.44 ± 0.0033	0.1 ± 0.0038	0.05 ± 0.0043	1.83 ± 0.0037	0.07 ± 0.0050	0.89
	70	0.63 ± 0.0033	0.44 ± 0.0043	0.1 ± 0.0042	0.05 ± 0.0044	1.83 ± 0.0040	0.07 ± 0.0043	0.88
	80	0.63 ± 0.0021	0.43 ± 0.0045	0.13 ± 0.0040	0.02 ± 0.0018	1.85 ± 0.0036	0.08 ± 0.0047	0.84
Ultrasonic power/W	240	0.63 ± 0.0023	0.46 ± 0.0044	0.12 ± 0.0039	0.02 ± 0.0022	1.84 ± 0.0045	0.07 ± 0.0040	0.87
	300	0.63 ± 0.0041	0.44 ± 0.0047	0.1 ± 0.0040	0.05 ± 0.0037	1.83 ± 0.0042	0.07 ± 0.0042	0.94
	360	0.7 ± 0.0042	0.47 ± 0.0035	0.08 ± 0.0036	0.01 ± 0.0046	1.93 ± 0.0042	0.06 ± 0.0036	0.79
	420	0.59 ± 0.0022	0.39 ± 0.0039	0.12 ± 0.0034	0.02 ± 0.0047	1.63 ± 0.0034	0.06 ± 0.0047	0.8
	480	0.62 ± 0.0043	0.38 ± 0.0040	0.12 ± 0.0037	0.02 ± 0.0034	1.63 ± 0.0047	0.04 ± 0.0041	0.76

**Table 3:** Independent factors and their levels.

Independent Factor	Levels		
	−1	0	1
A mL/g (liquid to material ratio)	10	20	30
B% (water content)	20	30	40
C min (extraction time)	30	40	50

**Table 4:** Response surface experiment results.

Serial Number	A/mL·g^−1^	B/%	C/min	Z
1	10	20	40	0.68
2	30	20	40	0.68
3	10	40	40	0.69
4	30	40	40	0.71
5	10	30	30	0.78
6	30	30	30	0.86
7	10	30	50	0.80
8	30	30	50	0.80
9	20	20	30	0.83
10	20	40	30	0.73
11	20	20	50	0.82
12	20	40	50	0.82
13	20	30	40	0.95
14	20	30	40	0.98
15	20	30	40	0.94
16	20	30	40	0.94
17	20	30	40	0.95

**Table 5:** Box–Behnken design with independent variables and measured response.

Source	Sum of		Mean	F	*p*-Value	
	Squares	df	Square	Value	Prob > F	
Model	0.16	9	0.018	21.91	0.0003	significant
A-Liquid to material ratio	0.00125	1	0.00125	1.51	0.2583	
B-Water content	0.00045	1	0.00045	0.54	0.4844	
C-Extraction time	0.0002	1	0.0002	0.24	0.6377	
AB	0.0001	1	0.0001	0.12	0.7381	
AC	0.0016	1	0.0016	1.94	0.2065	
BC	0.0025	1	0.0025	3.03	0.1254	
A^2^	0.067	1	0.067	80.96	< 0.0001	
B^2^	0.078	1	0.078	94.32	< 0.0001	
C^2^	0.001078	1	0.001078	1.31	0.2908	
Residual	0.00578	7	0.0008257			
Lack of Fit	0.0047	3	0.001567	5.8	0.0612	not significant
Pure Error	0.00108	4	0.00027			
Cor Total	0.17	16				
R^2^	0.9657					
Adj R^2^	0.9216					

**Table 6:** Standard curve of six compounds.

Analyte	Calibration Curve	Linearity Range (μg/μL)	R^2^	LOD	LOQ
isoliquiritin apioside	y = 5000,000x − 1202.6	0.0172–0.82	0.9996	0.00064	0.00194
liquiritin	y = 6000,000x − 1945.8	0.018–0.09	0.9997	0.00205	0.00621
Isoliquiritin	y = 9000,000x − 1581.9	0.0104–0.052	1	0.00029	0.00088
liquiritigenin	y = 10000,000x − 5257.6	0.014–0.07	1	0.00042	0.00128
glycyrrhizic acid	y = 80000,00x − 2242.8	0.0224–0.112	0.9999	0.00189	0.00573
glycyrrhetinic acid	y = 1000,000x − 3551.6	0.0088–0.044	0.9996	0.00225	0.00683

**Table 7:** The extraction rate and comprehensive score of each active ingredient of the verification experiment.

Serial Number	Extraction Rate/%						Z-Score
	Isoliquiritin Apioside	Liquiritin	Isoliquiritin	Liquiritigenin	Glycyrrhizic Acid	Glycyrrhetinic Acid	
1	0.66	0.46	0.12	0.11	1.84	0.021	0.92
2	0.65	0.46	0.12	0.11	1.81	0.021	0.92
3	0.66	0.45	0.12	0.11	1.87	0.021	0.93

**Table 8:** Different systems of DESs.

DESs Combination	HBAs	HBDs	Mol Ratio
DESs-1	ChCl	Glycol	1:2
DESs-2	ChCl	Glycol	1:3
DESs-3	ChCl	Glycol	1:4
DESs-4	ChCl	Glycol	1:5
DESs-5	ChCl	Glycol	1:6
DESs-6	ChCl	Glycerol	1:2
DESs-7	ChCl	Glycerol	1:3
DESs-8	ChCl	Glycerol	1:4
DESs-9	ChCl	Glycerol	1:5
DESs-10	ChCl	Glycerol	1:6
DESs-11	ChCl	1,3-Butanediol	1:2
DESs-12	ChCl	1,3-Butanediol	1:3
DESs-13	ChCl	1,3-Butanediol	1:4
DESs-14	ChCl	1,3-Butanediol	1:5
DESs-15	ChCl	1,3-Butanediol	1:6
DESs-16	ChCl	Lactic acid	1:2
DESs-17	ChCl	Lactic acid	1:3
DESs-18	ChCl	Lactic acid	1:4
DESs-19	ChCl	Lactic acid	1:5
DESs-20	ChCl	Lactic acid	1:6
DESs-21	ChCl	Urea	1:2
DESs-22	ChCl	Urea	1:3
DESs-23	ChCl	Malic acid	1:1
DESs-24	ChCl	Xylitol	1:1

## Data Availability

The Supplementary Materials are available online.

## Supplementary Materials

The following information is available. The influences of different chromatographic columns, different volumetric flows, and different column temperatures on relative correction factors are shown in Table S1, Table S2, and Table S3. Table S4 shows the results of comparison QASM and ESM.

## Sample Availability

Samples of the compounds are available from the authors.
