# Supplementary material for: Extraction and Analysis of Six Effective Components in Glycyrrhiza uralensis Fisch by Deep Eutectic Solvents (DES) Combined with Quantitative Analysis of Multi-Components by Single Marker (QAMS) Method
Source: Molecules. 2021 Mar 1;26(5):1310. doi: 10.3390/molecules26051310 (PMC7957616; doi:10.3390/molecules26051310)
Supplement: Supplementary file 1 [file molecules-26-01310-s001.pdf]

Supporting information

Extraction and Analysis of Six Effective Components in *Glycyrrhiza uralensis* Fisch by Deep Eutectic Solvents (DES) Combined with Quantitative Analysis of Multi-Components by Single Marker (QAMS) Method

Ping Yu [1,2,†], Qian Li [1,2,*,†], Yanmei Feng [1,2], Sinan Ma [1,2], Yuying Chen [1,2] and Guichen Li [2]

[1] College of Agronomy, Gansu Agricultural University, Lanzhou 730070, China; yp18325316756@163.com (P.Y.); fym15693446892@163.com (Y.F.); msn1805428551@163.com (S.M.); chenyuying0125@163.com (Y.C.)

[2] Gansu Provincial Key Laboratory of Aridland Crop Science,Lanzhou 730070, China; lguchen@163.com

* Correspondence: liqian1984@gsau.edu.cn

† These authors contributed equally to this work.

1. Repeatability of the correction factor

Three types of chromatographic columns were used to investigate the reproducibility of $f_{m/k}$ for the different components. The three types of columns are Symmetry C18 column, HederaODS-2 column and X-Peonyx AQ-C18 column. The results are shown in Table S1. At the same time, the influence of different column temperatures and different volumetric flow rates on the relative correction factors was also investigated. The results are shown in Table S2 and Table S3.

Table S1 The influence of different chromatographic columns on the relative correction factor

Column	$f_{\text{isoliquiritin apioside / glycyrrhizic acid}}$	$f_{\text{liquiritin/ glycyrrhizic acid}}$	$f_{\text{isoliquiritin / glycyrrhizic acid}}$	$f_{\text{liquiritigenin / glycyrrhizic acid}}$	$f_{\text{glycyrrhetinic acid / glycyrrhizic acid}}$
HederaODS-2	1.56	1.34	0.84	0.70	0.80
X-Peonyx AQ-C18	1.63	1.32	0.87	0.70	0.75
Symmetry C18	1.58	1.34	0.87	0.71	0.76
aveager	1.59	1.33	0.86	0.71	0.77
RSD	2.52%	0.95%	1.66%	1.08%	3.35%

Table S2 The influence of different volume flow on relative correction factor

Volume flow rate	$f_{\text{isoliquiritin apioside / glycyrrhizic acid}}$	$f_{\text{liquiritin/ glycyrrhizic acid}}$	$f_{\text{isoliquiritin / glycyrrhizic acid}}$	$f_{\text{liquiritigenin / glycyrrhizic acid}}$	$f_{\text{glycyrrhetinic acid / glycyrrhizic acid}}$
0.9ml/min	1.60	1.33	0.86	0.70	0.78
1.0ml/min	1.60	1.33	0.87	0.70	0.77
1.1ml/min	1.58	1.34	0.87	0.71	0.79
aveager	1.59	1.33	0.87	0.71	0.78
RSD	0.66%	0.50%	0.42%	1.15%	1.81%

Table S3 The influence of different column temperature on relative correction factor

column temperatures	$f_{\text{isoliquiritin apioside / glycyrrhizic acid}}$	$f_{\text{liquiritin/ glycyrrhizic acid}}$	$f_{\text{isoliquiritin / glycyrrhizic acid}}$	$f_{\text{liquiritigenin / glycyrrhizic acid}}$	$f_{\text{glycyrrhetinic acid / glycyrrhizic acid}}$
30℃	1.60	1.35	0.88	0.70	0.77
32℃	1.61	1.33	0.86	0.70	0.77
25℃	1.58	1.34	0.88	0.71	0.78
aveager	1.59	1.34	0.87	0.71	0.77
RSD	0.80%	0.82%	1.10%	1.35%	0.52%

2. (QAMS) and external standard method (ESM) measurement results comparison

6 batches of *Glycyrrhiza uralensis* Fisch powder was taken to carried out the experiments. The content of the six flavonoids in the samples determined by QASM and ESM were compared, and the error was expressed by the relative error (RE). RE% = [(QAMS - ESM)/EMS] *100%. The results are shown in Table S4 The relative average deviation of the content of each component measured by the two methods is less than 5%, which indicates that the method is reliable.

Table S4 The results of QASM and ESM

Sample Source	isoliquiritin apioside		RE/%	liquiritin		RE/%	isoliquiritin		RE/%	liquiritigenin		RE/%	glycyrrhizic acid	glycyrrhetinic acid		RE/%
	ESM	QASM		ESM	QASM		ESM	QASM		ESM	QASM		ESM	ESM	QASM	
S1	1.96	1.98	-0.65	0.95	0.95	-0.40	0.18	0.17	-2.30	0.59	0.58	-1.72	1.65	0.07	0.07	-3.48
S2	1.65	1.67	-0.65	1.24	1.24	-0.40	0.20	0.19	-2.30	0.41	0.41	-1.01	1.75	0.05	0.05	-3.48
S3	0.55	0.55	-0.65	0.83	0.82	-0.40	0.07	0.07	-2.30	0.33	0.33	-1.01	0.84	0.04	0.04	-3.48
S4	0.66	0.67	-0.65	0.46	0.46	-0.40	0.10	0.10	-2.30	0.07	0.07	-1.01	1.69	0.02	0.02	-3.48
S5	2.60	2.62	-0.65	9.29	9.25	-0.40	0.74	0.72	-2.30	0.83	0.82	-1.01	2.86	0.00	0.00	-3.48
S6	2.51	2.53	-0.65	0.74	0.74	-0.40	0.69	0.68	-2.30	0.20	0.20	-1.01	2.26	0.10	0.09	-3.48
